# Rehabilitation of a Patient With D12 Wedge Compression Fracture and Bilateral Foot Drop With Spinal Fusion and Posterior Decompression: A Case Report

**DOI:** 10.7759/cureus.51561

**Published:** 2024-01-03

**Authors:** Shruti S Bhoge, Vrushali Athawale, Tejaswini Fating

**Affiliations:** 1 Community Health Physiotherapy, Ravi Nair Physiotherapy College, Datta Meghe Institute of Higher Education and Research, Wardha, IND

**Keywords:** contract-relax, foot drop, vertebral wedge compression fracture, posterior decompression, spinal fusion, treatment, physiotherapy, case report, osteoporotic vertebral compression fracture (ovcf), vertebral fracture

## Abstract

Vertebral fracture (VF) is one of the most common injuries seen in individuals with osteoporosis, especially in post-menopausal females. There is an increase in bone resorption rate, leading to the destruction of the microarchitecture of bone. A 67-year-old female patient diagnosed with wedge compression fracture of the D12 vertebra, mild compression of the spinal cord, and bilateral foot drop came to a tertiary care hospital, where she underwent spinal fusion at the D11-L1 level and posterior decompression, after which she was referred to physiotherapy, where a patient-tailored treatment protocol was made and implemented over three weeks. Outcome measures like the visual analog scale (VAS), functional independence measure (FIM), and Oswestry's low back disability questionnaire were recorded before and after rehabilitation, and improvement in pain and activities of daily living (ADL) was found. The patient needed mild assistance. There was also improvement in the range and strength of the lower limb muscles. This case report aims to provide a comprehensive treatment protocol for a post-operative spinal fusion and bilateral foot drop patient.

## Introduction

Vertebral fracture (VF) is a common phenomenon in osteoporotic patients, especially in post-menopausal women [1]. Primary osteoporosis is commonly evident a decade after menopause. There is an increase in osteoclastic activity, i.e., bone resorption rate [2]. The bone mineral density decreases, and the bone becomes brittle due to the deterioration of bone architecture. The most common fractures in these women are of the hip and vertebra [3]. Vertebral fractures are more common in the mid-thoracic and thoracolumbar regions in osteoporotic patients. There are three types of VF, namely biconcave, compression, and wedge. Wedge VFs (50%) are the most common type of fracture [4]. According to a study conducted in 2020, annually, out of every 1000 women, approximately 11 women suffer from a vertebral compression fracture (VCF), which is double the number of men suffering from VCF. It is estimated that a quarter of post-menopausal women suffer from VCF [5]. The prevalence of VFs in North America and Asia is found to be highest [6]. These fractures are mostly diagnosed with X-rays, and injuries to the spinal cord are diagnosed with MRI [7].

Surgery is decided based on the type of fracture, degree of comminution, and neurological involvement. Minimally invasive procedures involve kyphoplasty and vertebroplasty, which involve injecting cement (polymethylmethacrylate) into the fractured vertebra [8, 9]. Spinal fusion is an invasive procedure that uses pedicle screws and connecting rods for spinal stabilization. The most common approach used for this surgery is the posterior approach for interbody fusion, which is found to have a successful fusion rate and fewer complications, but nowadays, anterior and lateral approaches are also being used [10].

In VFs, there is a high chance of retropulsion of fragments of the vertebra into the spinal canal, potentially compromising the spinal cord by narrowing the spinal canal. Due to spinal canal narrowing or spinal cord compression, a wide variety of neurological manifestations can be seen [11]. There is weakness or paralysis of muscles, loss of sensations, tingling sensations, radiating pain, diminished or absent reflexes, etc. These manifestations are present below the level of compression of the cord. Foot drop is another one of the manifestations of spinal cord compression at or above the L5 level. The incidence of foot drop in lumbar spinal stenosis was found to be 5%-12% [12]. After surgery, physiotherapy becomes of paramount importance. The goals of physiotherapy after spinal fusion surgery are to prevent secondary complications, prevent disability, improve tone and power of muscles, improve mobility of the spine and joints, improve balance and gait, improve quality of life (QoL), etc. [13]. The physiotherapy is found to be successful in improving balance and lower limb muscle strength through a strengthening and balance exercise protocol in vertebral fracture patients [14]. The exercise protocols also proved to be beneficial in improving bone mineral density in osteoporotic patients as well as quality of life [15]. In this case report, we see the case of a 67-year-old female with a D12 fracture and bilateral foot drop, treated with spinal fusion and posterior decompression, and how physical therapy contributed to her improvement.

## Case presentation

Patient information

A 67-year-old female patient fell on her back one and a half months ago. She developed pain in her mid-back region. The pain was sudden in onset, dull, aggravated during back movements, and relieved on medication. For this complaint, she visited a local hospital, where she was given analgesics for her pain. But the discomfort persisted, and she also developed radiating pain, tingling sensations in both lower limbs, and difficulty walking, for which she visited another healthcare center where an MRI and X-ray were done. The investigations revealed a wedge compression fracture of the D12 vertebra and mild compression of the spinal cord. She came to Acharya Vinoba Bhave Rural Hospital, where she was offered surgery for the same. She underwent spinal fusion at the D11-L1 level and posterior decompression. After one week, the patient was referred for physiotherapy with chief complaints of pain at the suture site, difficulty walking, an inability to lift the feet, and difficulty doing activities of daily living (ADL). She had no relevant medical history.

Clinical findings

After obtaining consent from the patient, the examination was initiated. The patient was conscious, cooperative, and oriented to place, person, and time. On observation, both ankles were in an ankle-foot orthosis. She rated her pain at 6.3 on the visual analog scale (VAS). Her pain was dull and aching, present over the suture site on her back, roughly over the thoracolumbar region of the back. It aggravated during movement and was relieved with rest and medication. On examination, there was a spasm of paraspinal muscles. Tenderness was present around the suture site, which was grade 2 (the patient complained of pain and winced). The power of dorsiflexors was found to be zero, which refers to no contraction, as shown in Table 1.

**Table 1:** Pre-rehabilitation findings of MMT, a week after the surgery MMT: manual muscle testing 0: No contraction; 2: Full range of motion in gravity-eliminated position; 3: Full range of motion against gravity position; 4: Full range of motion against minimal resistance; 5: Full range of motion against maximal resistance

Joints	Muscle groups	Right	Left
Hip	Flexors	3/5	3/5
	Extensors	3/5	3/5
Knee	Flexors	4/5	4/5
	Extensors	4/5	4/5
Ankle	Dorsiflexors	0/5	0/5
	Plantarflexors	2/5	2/5

The range of motion (ROM) of the ankle was not assessable due to no contraction of muscles (Table 2).

**Table 2:** Pre-rehabilitation findings of ROM, a week after the surgery ROM: range of motion; N/A: not applicable

Joints	Movement	Normal	Right	Left
Hip	Flexion	0˚-120˚	0˚-100˚	0˚-104˚
	Extension	0˚-30˚	0˚-22˚	0˚-26˚
Knee	Flexion	0˚-135˚	0˚-118˚	0˚-125˚
Ankle	Plantarflexion	0˚-50	N/A	N/A
	Dorsiflexion	0˚-20˚	N/A	N/A

On reflex examination, the ankle jerk on both sides was found to be diminished. The sensory examination revealed normal findings. The functional independence measure score was found to be 46, which means the patient needs maximal assistance to perform her day-to-day tasks. On the Oswestry low back disability questionnaire, the patient had a score of 26, which means the patient is severely disabled.

Investigations

**Figure 1:**
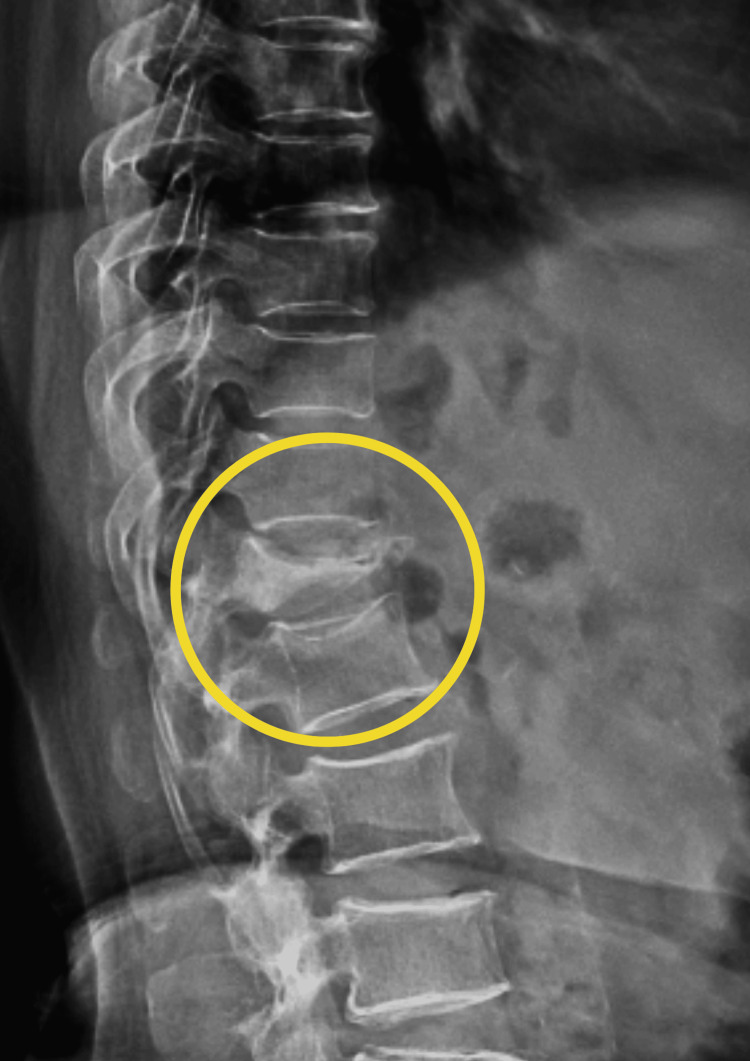
Pre-operative X-ray of the spine The yellow arrow shows a D12 vertebra wedge compression fracture.

The patient underwent an X-ray and an MRI. X-rays of the spine revealed a wedge compression fracture of the D12 vertebra, as shown in Figure 1.

**Figure 2:**
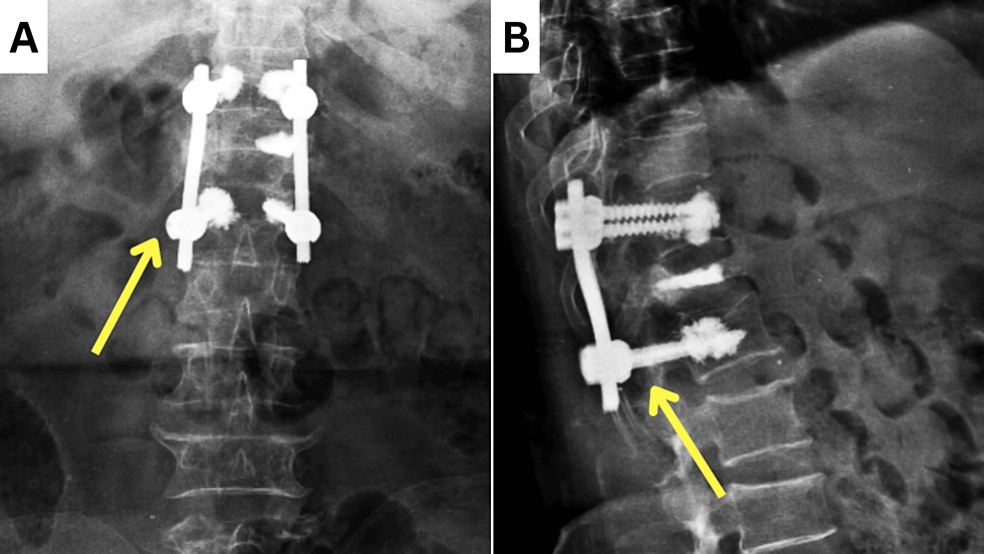
Post-operative X-ray of the spine The yellow arrow depicts the pedicle screw and connecting rods attaching D11 to the L1 vertebra. A: anterior view; B: lateral view

The MRI showed a complete collapse of the D12 vertebral body with retropulsion, causing moderate spinal canal narrowing and mild compression of the spinal cord. It also revealed mild spinal canal narrowing at L2-L3, L3-L4, and L5-S1 levels and severe canal narrowing at L4-L5 levels. The post-operative X-ray showed pedicle screws attached to the D11 and L1 vertebrae, which were connected by connecting rods (Figure 2).

Therapeutic intervention

The patient was referred to physiotherapy one week after surgery. The patient received physiotherapy treatment for three weeks, after which she was discharged. During bedside sitting and walking, the patient was compulsorily wearing a Taylor brace to stabilize the spine. The physiotherapy protocol is mentioned in Table 3.

**Table 3:** Physiotherapy protocol followed over a period of three weeks TENS: transcutaneous electrical nerve stimulation; secs: seconds; AROM: active range of motion; PROM: passive range of motion; kg: kilogram; D1: diagonal pattern; N/A: not applicable

Goals	Intervention	Regimen
Patient education	The patient was informed of her condition, the surgery performed, and the importance of physiotherapy treatment. She was also made aware of the progression of treatment and precautions to be taken.	N/A
To relieve pain	Cryotherapy was given at the pain site.	For seven minutes, twice a day
	TENS was placed around the suture site.	For 20 minutes
To improve the strength of abdominal muscles and back muscles	Static back and static abdominal contractions	10 repetitions × 1 set with 10 seconds of hold, progressing to 30 seconds of hold
To improve bed mobility	Teaching the patient to roll from supine to side lying to sitting position.	Every two hours
To improve the range of motion of lower limb joints	AROM exercises to the hip and knee and PROM exercises to the ankle joint	10 repetitions × 1 set for each movement
To improve the strength of the muscles of the lower limbs	Static strengthening of the gluteals, hamstrings, and quadriceps muscles, and then progressing to dynamic strengthening using a one-kg weight cuff	10 repetitions × 1 set with 10 seconds of hold for static strengthening and 10 repetitions × 1 set for dynamic strengthening
To improve the strength of ankle dorsiflexors	Faradic stimulation of the extensor hallucis longus and extensor digitorum longus muscles.	90 contractions in one session. The contractions were divided into three sets of 30 contractions, followed by a one-minute rest
	After the muscle regained power, we included rhythmic initiation for the ankle joint in the D1 flexion pattern, concentrating on the ankle dorsiflexion movement. Further, we progressed to the contract-relax technique to improve the strength of dorsiflexors.	4 repetitions × 1 set for rhythmic initiation and 10 repetitions × 3 sets of contract-relax
To improve gait	The patient was using moderate assistance from a walker for walking, which progressed to using a tripod cane, i.e., minimal support during walking.	N/A
To prevent secondary complications	Breathing exercises to prevent respiratory infections and bed mobility exercises to prevent bed sores	10 reps × 1 set for breathing exercises

**Figure 3:**
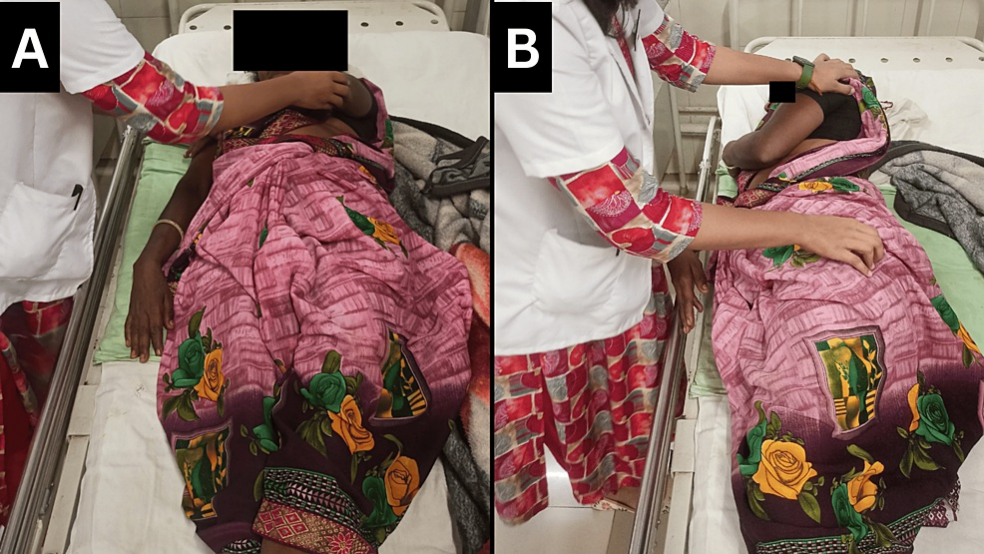
Bed mobility exercises A: supine; B: side lying

**Figure 4:**
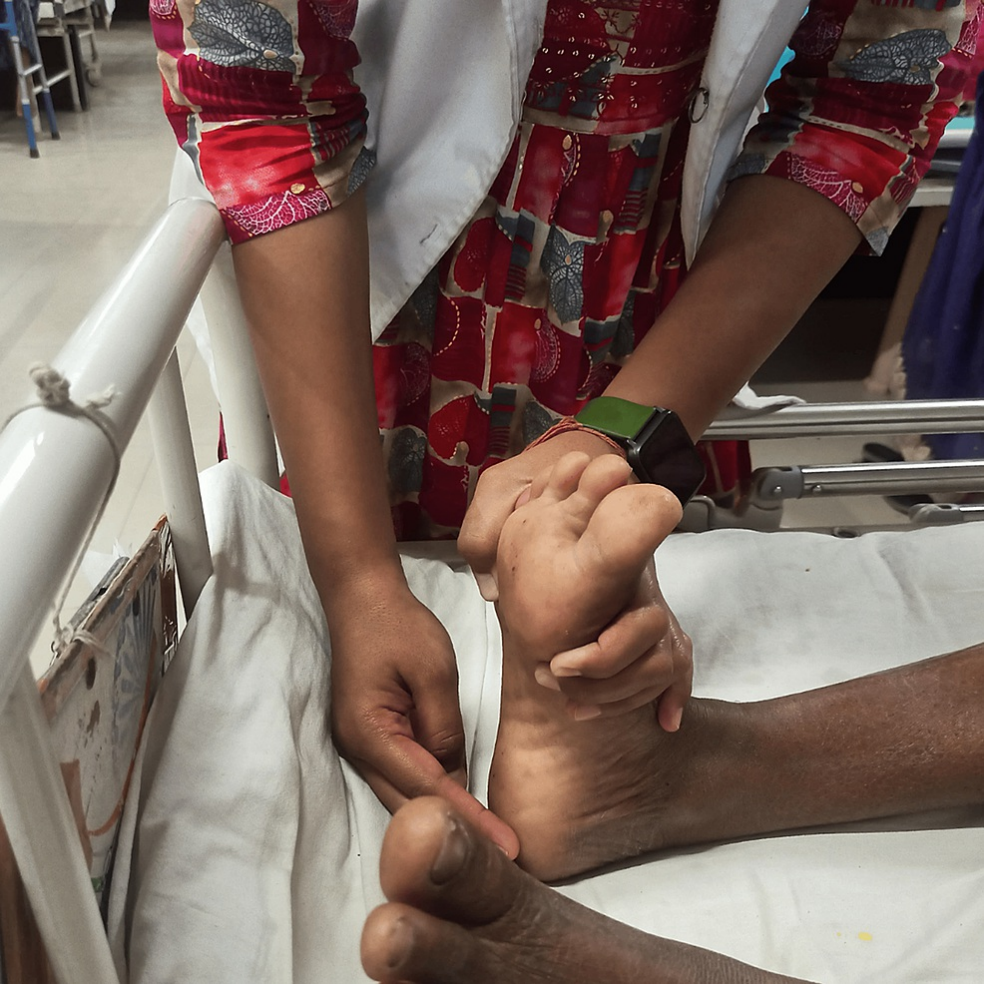
Contract-relax technique for dorsiflexors of the ankle

Figures 3-4 show the patient receiving physiotherapy treatment.

Outcome measures

After three weeks of physiotherapy treatment, post-rehabilitation findings were recorded. Outcome measures like the VAS, functional independence measure (FIM), and Oswestry low back disability questionnaire were recorded before and after rehabilitation. We found improvement in pain and ADL, and the patient needed minimal assistance, as mentioned in Table 4.

**Table 4:** Pre- and post-rehabilitation findings of outcome measures VAS: visual analogue scale; FIM: functional independence measure

Outcome measures	Pre-rehabilitation	Post-rehabilitation
VAS	6.3	1.7
FIM	46 (maximal assistance)	73 (minimal assistance)
Oswestry low back disability questionnaire	26 (severe disability)	12 (mild disability)

There was also improvement in the ROM (Table 5) and the strength of the lower limb muscles (Table 6).

**Table 5:** Pre and post-rehabilitation findings of MMT MMT: manual muscle testing 0: No contraction; 2: Full range of motion in gravity-eliminated position; 3: Full range of motion against gravity position; 3: Initiates some but not complete range of motion against gravity position; 4: Full range of motion against minimal resistance; 5: Full range of motion against maximal resistance

MMT		Pre-rehabilitation		Post-rehabilitation	
Joints	Muscle groups	Right	Left	Right	Left
Hip	Flexors	3/5	3/5	4/5	4/5
	Extensors	3/5	3/5	4/5	4/5
Ankle	Dorsiflexors	0/5	0/5	3-/5	3-/5
	Plantarflexors	2/5	2/5	3/5	3/5

**Table 6:** Pre and post-rehabilitation findings of ROM ROM: range of motion; N/A: not applicable

ROM		Pre-rehabilitation		Post-rehabilitation	
Joints	Movement	Right	Left	Right	Left
Hip	Flexion	0˚-100˚	0˚-104˚	0˚-110˚	0˚-115˚
	Extension	0˚-52˚	0˚-56˚	0˚-58˚	0˚-60˚
Knee	Flexion	0˚-118˚	0˚-125˚	0˚-125˚	0˚-130˚
Ankle	Plantarflexion	N/A	N/A	0˚-45˚	0˚-48˚
	Dorsiflexion	N/A	N/A	0˚-10˚	0˚-15˚

The ankle jerk was found to improve from a diminished to a normal reflex when assessed after three weeks of treatment.

## Discussion

In the above case, we saw the rehabilitation of a post-spinal fusion patient who was also having bilateral foot drops. Our patient-tailored physiotherapy protocol showed immense progress after three weeks of treatment. The literature on physiotherapy intervention in vertebral wedge compression fractures is very limited. This patient also had a bilateral foot drop due to compression of the spinal cord. In this study, we used active ROM exercises for the hip and knee as well as passive ROM exercises for the ankle; we found improved ranges of hip, knee, and ankle. Burile et al. used active-assisted ROM exercises for the upper limb in cervical myelopathy treated with spinal fusion but found no improvement in the range of the upper limb [16]. We also used faradic current stimulation of the ankle dorsiflexor muscles, which led to a significant improvement in the strength of those muscles. Kachhwani et al. used functional electrical stimulation for ankle dorsiflexors as well as plantarflexor muscles and recorded significant improvement in ankle function in a D11 traumatic fracture patient [17]. Bed mobility exercises were also incorporated into the protocol, as they are pertinent to avoid further injury to the body due to improper body mechanics [18].

We also included proprioceptive neuromuscular facilitation (PNF) techniques in our protocol to improve the strength of ankle dorsiflexors, specifically rhythmic initiation, and contract-relax. The addition of the PNF technique showed improved range and strength of ankle dorsiflexion. Nakada et al. also employed PNF patterns like chopping and lower limb diagonal patterns in a patient with foot drop due to demyelinating polyneuropathy and found enhanced range and strength of ankle dorsiflexor muscles [19]. This technique helps in not only improving the strength of the muscles but also the range of a joint [20].

## Conclusions

Vertebral fractures are one of the most prevalent fractures in women, especially in the post-menopausal phase. A lot of the time, this fracture goes unnoticed, but when this fracture causes severe manifestations eventually affecting ADLs and QoL, they need to be dealt with as soon as possible. In this case report, we saw a postmenopausal female with a D12 vertebral wedge compression fracture and foot drop; she was later treated with spinal fusion and was referred to the physiotherapy department, where she received a patient-tailored physiotherapy treatment. The patient had improved range and strength of the muscles of the ankle joint and spine, ADLs, a reduced disability index, and improved functional independence. As there is little literature on the exact treatment of this type of case, this case report adds a piece of vital information to already available information.
